# Ionic liquid accelerates the crystallization of Zr-based metal–organic frameworks

**DOI:** 10.1038/s41467-017-00226-y

**Published:** 2017-08-02

**Authors:** Xinxin Sang, Jianling Zhang, Junfeng Xiang, Jie Cui, Lirong Zheng, Jing Zhang, Zhonghua Wu, Zhihong Li, Guang Mo, Yuan Xu, Jinliang Song, Chengcheng Liu, Xiuniang Tan, Tian Luo, Bingxing Zhang, Buxing Han

**Affiliations:** 1 Beijing National Laboratory for Molecular Sciences, CAS Key Laboratory of Colloid, Interface and Chemical Thermodynamics, CAS Research/Education Center for Excellence in Molecular Sciences, Institute of Chemistry, Chinese Academy of Sciences, Beijing, 100190 People’s Republic of China; 2 School of Chemistry and Chemical Engineering, University of Chinese Academy of Sciences, Beijing, 100049 People’s Republic of China; 3 Beijing Synchrotron Radiation Facility, Institute of High Energy Physics, Chinese Academy of Sciences, Beijing, 100049 People’s Republic of China

## Abstract

The Zr-based metal–organic frameworks are generally prepared by solvothermal procedure. To overcome the slow kinetics of nucleation and crystallization of Zr-based metal–organic frameworks is of great interest and challenging. Here, we find that an ionic liquid as solvent can significantly accelerate the formation of Zr-based metal–organic frameworks at room temperature. For example, the reaction time is shortened to 0.5 h in 1-hexyl-3-methylimidazolium chloride for Zr-based metal–organic framework formation, while that in the conventional solvent N,N-dimethylformamide needs at least 120 h. The reaction mechanism was investigated in situ by ^1^H nuclear magnetic resonance, spectroscopy synchrotron small angle X-ray scattering and X-ray absorption fine structure. This rapid, low-energy, and facile route produces Zr-based metal–organic framework nanoparticles with small particle size, missing-linker defects and large surface area, which can be used as heterogeneous catalysts for Meerwein–Ponndorf–Verley reaction.

## Abstract (web summary)

Crystallization kinetics of metal-organic frameworks in conventional organic solvents are usually very slow. Here, the authors show that an ionic liquid medium accelerates considerably the formation of Zr-based metal-organic frameworks that are active catalysts in the Meerwein-Ponndorf-Verley reaction.

## Introduction

Metal–organic frameworks (MOFs) are a class of porous materials that have been studied extensively due to their wide variety of applications such as gas storage and separation^1^, catalysis^2^, energy storage^3^, light harvesting^4^, etc. Among the diverse kinds of MOFs that have been investigated, Zr-based MOFs have grown in popularity because of their excellent thermal, aqueous, acid stability, and easy modification with a desired functionality compared to other common MOFs^5^. The Zr-based MOFs are generally prepared by solvothermal procedure, which needs a heating over 100 °C and long reaction time up to days. How to overcome the slow kinetics of nucleation and crystallization of MOFs is a very interesting topic^6^. Some efforts have been devoted to accelerate the formation of Zr-based MOFs, mainly including energy input method (e.g., microwave synthesis^7^) and adding additives (e.g., monocarboxylic acid^8, 9^ and cosolvent^10^). In comparison with high-energy process, the utilization of intrusive stimulus at room temperature is more desirable. However, the additives will inevitably cause the contamination to the product and the post-process for additive removing is complex. To develop facile, rapid, low-energy, and environmentally benign routes for the synthesis of Zr-based MOFs is much attractive.

Ionic liquids (ILs) have received increasing attention due to their peculiar properties such as lack of measurable vapour pressure, high ionic conductivity and polarity, designable structure and tunable properties^11^. ILs have been known as solvents for MOFs synthesis (ionothermal synthesis) for more than a decade^12–17^. In comparison with the solvothermal procedure by using the conventional solvents (water and organic solvents), the ionothermal synthesis for MOFs has many advantages. Most importantly, ILs can provide diverse reaction conditions owing to their designable and tunable characteristics. During the ionothermal synthesis, IL can act not only as solvent but also as structure-directing agent because the liquid is highly structured with relatively long-range correlations. Such unique features have been utilized for modulating the structures of MOFs, e.g., inducing a chiral effect in the ionothermal synthesis of MOFs^18–21^. Moreover, IL as a medium for MOF synthesis has many other appealing properties such as the ability to simultaneously dissolve both organic and inorganic precursors, high thermal, and chemical stability and extremely low volatility. It is very meaningful to utilize IL as a solvent for promoting MOF crystallization at room temperature in view of the ionic and organic compositions of IL, which would interact with MOF precursors to influence MOF formation.

Here, we report our finding that an aprotic IL [C_*n*_mim]X (X = Cl^−^, Br^−^, and I^−^) as solvent can significantly accelerate the room-temperature formation of Zr-based MOFs. The reaction time is extremely shortened to 0.5 h in 1-hexyl-3-methylimidazolium chloride ([Hmim]Cl), while the room-temperature synthesis in the most widely adopted solvent N,N-Dimethylformamide (DMF) needs at least 120 h. The in situ small angle X-ray scattering (SAXS), X-ray absorption fine structure (XAFS), and ^1^H nuclear magnetic resonance (^1^H NMR) spectroscopy were applied to dynamically trace the forming process of MOF nanoparticles in IL. The rapid and room-temperature synthetic route produces Zr-based MOF nanoparticles with small particle size, missing-linker defects, and large surface area, which have shown potential for catalyzing Meerwein–Ponndorf–Verley (MPV) reaction.

## Results

### Rapid crystallization of UiO-66 in [Omim]Cl

**Fig. 1:**
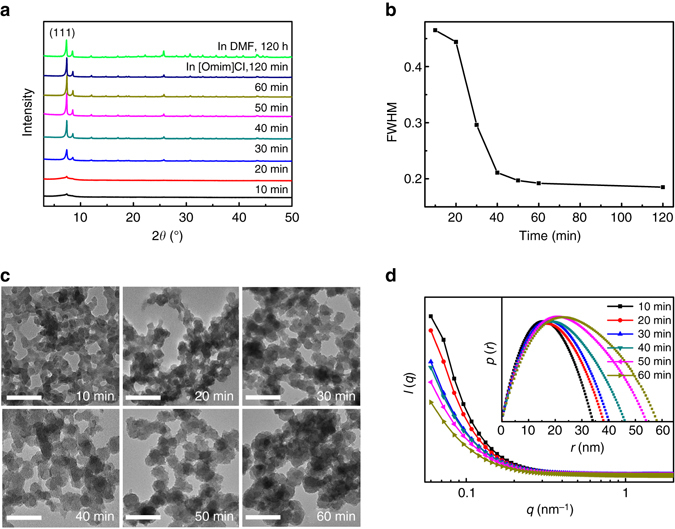
Crystallization of UiO-66 in [Omim]Cl with reaction time. **a** XRD patterns of the products synthesized in [Omim]Cl with different reaction time and in DMF with reaction time of 120 h. **b** The full-width half-maximum *FWHM* of the (111) reflection of the products synthesized in [Omim]Cl against reaction time. **c** TEM images of the products synthesized in [Omim]Cl with different reaction time. Scale bar, 200 nm. **d** In situ SAXS profiles and pair-distance distribution function curves (the inset) of the reaction systems for UiO-66 synthesis in [Omim]Cl at different reaction time

UiO-66 (UiO stands for University of Oslo) is a prototypical Zr-based MOF with the formula Zr_6_O_4_(OH)_4_(BDC)_6_ (BDC = 1,4-benzene-dicarboxylate) and has potential applications in a variety of fields^5^. Here UiO-66 was synthesized in 1-octyl-3-methylimidazolium chloride ([Omim]Cl) at room temperature by the reaction of dichlorooxozirconium (ZrOCl_2_·8H_2_O) and terephthalic acid (H_2_BDC) with the help of acetic acid (HAc), which is usually used in UiO-66 synthesis as a modulator^5^. As the reaction time is as short as 1 h, the X-ray diffraction (XRD) peak positions and relative intensities of the solid product synthesized in [Omim]Cl agree well with those of the reported UiO-66 (Fig. 1a)^22^. In sharp contrast, no precipitate was obtained in DMF even as the reaction time was prolonged to 96 h and at least 120 h is needed for the UiO-66 formation at room temperature (Fig. 1a). Obviously, [Omim]Cl as a solvent significantly promotes the formation of UiO-66 at room temperature as compared with the conventional organic solvent. The full-width half-maximum (FWHM) of (111) peak of the UiO-66 synthesized in [Omim]Cl declines within 50 min and keeps nearly unchanged thereafter (Fig. 1b), indicating the increased crystallinity and larger nanocrystallites with prolonged reaction time. The rapid formation of UiO-66 in [Omim]Cl was further confirmed by FT-IR spectrum and X-ray photoelectron spectroscopy (Supplementary Figs. 1 and 2). By element analysis (Supplementary Fig. 3), the formula of the as-synthesized UiO-66 can be defined to be Zr_6_O_4_(OH)_4_(BDC)_5.1_. The presence of missing-linker defects (i.e., open metal sites) in the UiO-66 was also supported by thermogravimetric analysis (TGA)^23–25^ (Supplementary Fig. 4). The missing-linker defects of the as-synthesized UiO-66 may result from the low synthesis temperature and rapid crystallization^26^. The UiO-66 has a surface area of 1519 m^2^ g^−1^, as determined by N_2_ adsorption-desorption measurement (Supplementary Fig. 5), which is higher than that produced by solvothermal process in DMF (1187 m^2^ g^−1^)^22^.

The solid products obtained from [Omim]Cl at different reaction time were characterized by transmission electron microscopy (TEM). When the reaction time is as short as 10 min, the product exhibits a cross-linked morphology (Fig. 1c). With the prolonged reaction time, the particles are more discrete with each other and grow larger. At 1 h, the crystallined UiO-66 forms with particle size of about 80 nm. To get more information on the growth of UiO-66 crystals in [Omim]Cl, the reaction system for UiO-66 synthesis in [Omim]Cl was investigated in situ by SAXS, which is a useful technique to study the colloid systems in their native and solution environments^27^. The relative intensity (*I*) of SAXS curves shifts to low scattering wavevector (*q*) with increasing reaction time (Fig. 1d), indicating that the nanoparticles grow larger. From the pair-distance distribution function *p*(*r*) curves (inset in Fig. 1d), the sizes of particles in the reaction system at 10, 20, 30, 40, 50, and 60 min are 34, 38, 40, 46, 54, and 58 nm, respectively. The particle sizes are a little smaller than those of the separated solid particles characterized by TEM images, which can be attributed to the particle growth in separation and drying process.

### Versatility and tunability of ILs for UiO-66 synthesis

ILs are designable solvents and their structures and properties can be tuned by adjusting the cations and anions. Here we explored the modulation on UiO-66 crystallization by changing the cation and anion of IL. First, 1-hexyl-3-methylimidazolium chloride ([Hmim]Cl) and 1-decyl-3-methylimidazolium chloride ([Dmim]Cl) were employed. The crystallized UiO-66 can be obtained rapidly at 0.5 h and 2 h in [Hmim]Cl and [Dmim]Cl, respectively (Supplementary Figs. 6 and 7), i.e., the MOF crystallization is promoted in the IL with shorter alkyl chain. The average sizes of the UiO-66 nanoparticles synthesized in [Hmim]Cl and [Dmim]Cl are 60 and 100 nm, respectively (Supplementary Fig. 8). Second, the ILs with the same cation 1-octyl-3-methylimidazolium ([Omim]^+^) and different anions including Br^−^ and I^−^ were employed. The time for UiO-66 crystallization is prolonged to 6 h and 18 h for [Omim]Br and [Omim]I, respectively (Supplementary Fig. 9).

Furthermore, the Zr-based MOFs with different chemical compositions and functionalities were synthesized in [Omim]Cl at room temperature, including UiO-66-NO_2_, UiO-66-NH_2_, UiO-66-OH, UiO-67 (biphenyl-4,4′-dicarboxylate as linker), and Zr-MOF-bpydc (bpydc = 2,2′-bipyridine-5,5′-dicarboxylic acid). The minimum time that is needed for the crystallization of the above five MOFs is 6, 18, 24, 24, and 32 h, respectively (Supplementary Figs. 10 and 11). By comparison with the synthesis for non-functionalized UiO-66, it is evident that either the presence of substituent on H_2_BDC or the extension of the organic linker slows down the crystallization rate.

### In situ NMR spectra for UiO-66 formation in IL

**Fig. 2:**
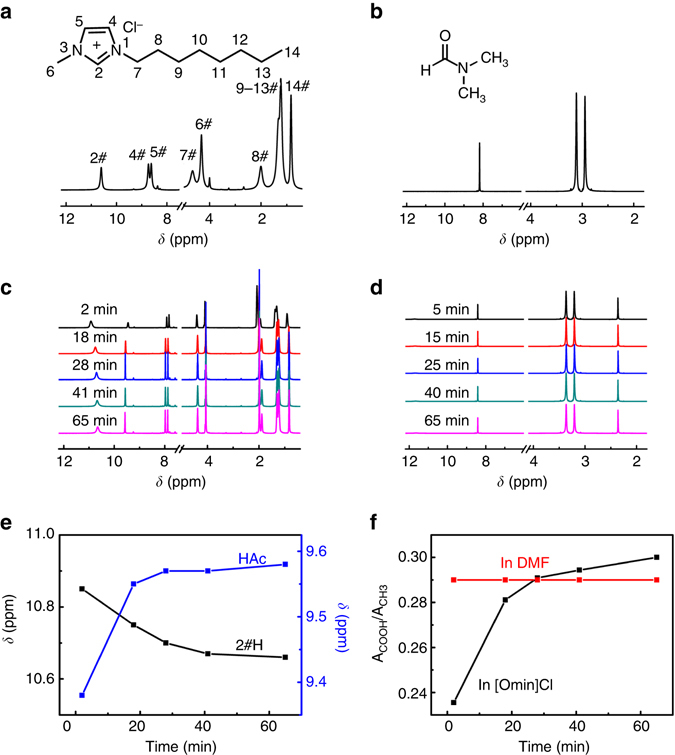
^1^H NMR spectra of [Omim]Cl, DMF and UiO-66 synthesis mixtures. **a**, **b** Molecular structures and ^1^H NMR spectra of [Omim]Cl and DMF, respectively. **c**, **d** In situ ^1^H NMR spectra of the UiO-66 synthesis mixtures in [Omim]Cl and DMF at 298.2 K, respectively. **e** Chemical shift of 2#H in [Omim]Cl and active H in HAc of the UiO-66 synthesis mixtures in [Omim]Cl with reaction time. **f** A_COOH_/A_CH3_ of the UiO-66 synthesis mixtures in [Omim]Cl and DMF, respectively

The above results prove that [Omim]Cl can significantly accelerate the formation of crystallined UiO-66 at room temperature. The ^1^H NMR spectra of the reaction system for UiO-66 synthesis in [Omim]Cl were investigated in situ. Upon reaction, the ^1^H NMR spectra of the reaction systems in [Omim]Cl (Fig. 2c) show a large difference with that of pure [Omim]Cl (Fig. 2a), while those of the reaction systems in DMF are nearly identical to that of pure DMF except the occurrence of peaks resulting from modulator HAc (Fig. 2b, d). The chemical shift (δ) of 2#H down-field shifts from 10.61 ppm in pure [Omim]Cl to 10.85 ppm for the reaction system in [Omim]Cl at reaction time of 2 min, indicative of the formation of C-H···O hydrogen bond between 2#H and the oxygen in reaction system^28, 29^. With prolonged reaction time, the δ of 2#H shifts toward the shielded direction and approaches that of pure [Omim]Cl at 1 h (Fig. 2e). It indicates that the hydrogen bond C-H···O is weakened with reaction time until an equilibrium is achieved. No obvious changes for other H of [Omim]Cl were observed with reaction time (Fig. 2c).

Along with the change of the δ of 2#H of [Omim]Cl in the reaction process, the δ of the active hydrogen in HAc shifts obviously. At reaction time of 2 min, the δ of active hydrogen of HAc occurs at 9.45 ppm (Fig. 2c), which is much lower than that of pure HAc (11.42 ppm). It means that there are strong interactions between HAc and the reaction system. With prolonged reaction time, the δ moves to higher field gradually, opposite to the change of 2#H of [Omim]Cl (Fig. 2e). To get a further information, the integral peak area ratios between the active H of COOH and H of methyl in HAc (A_COOH_/A_CH3_) for the reactions in [Omim]Cl and DMF were analyzed, respectively (Fig. 2f). At the initial reaction stage, the A_COOH_/A_CH3_ in [Omim]Cl is much lower than that in DMF, confirming the strong interactions between HAc and [Omim]Cl. It is easily understood that as a potential electron-pair donor, carboxylic acid can form intermolecular hydrogen bonds with imidazolium ring of [Omim]Cl. Subsequently, the A_COOH_/A_CH3_ in [Omim]Cl increases rapidly within half an hour and exceeds that in DMF thereafter, while that in DMF keeps unchanged. It has been reported that the framework construction proceeds through an exchange between modulator HAc and linker H_2_BDC at the coordination sites of zirconium ion^30^. The large increase of A_COOH_/A_CH3_ indicates that the exchange rate is greatly accelerated with the assistance of [Omim]Cl, thus promoting the UiO-66 construction. The in situ ^1^H NMR spectra of UiO-66 formation in [Omim]Cl manifest that a coordination modulation mechanism seems quite possible, i.e., [Omim]Cl and HAc accelerate the UiO-66 formation cooperatively. The results of ^1^H NMR spectra for the UiO-66 synthesized in different ILs (Supplementary Figs. 12 and 13) are similar to that for the UiO-66 synthesis in [Omim]Cl, implying that the UiO-66 undergoes the same crystallization procedure.

### In situ XAFS for UiO-66 formation in IL

**Fig. 3:**
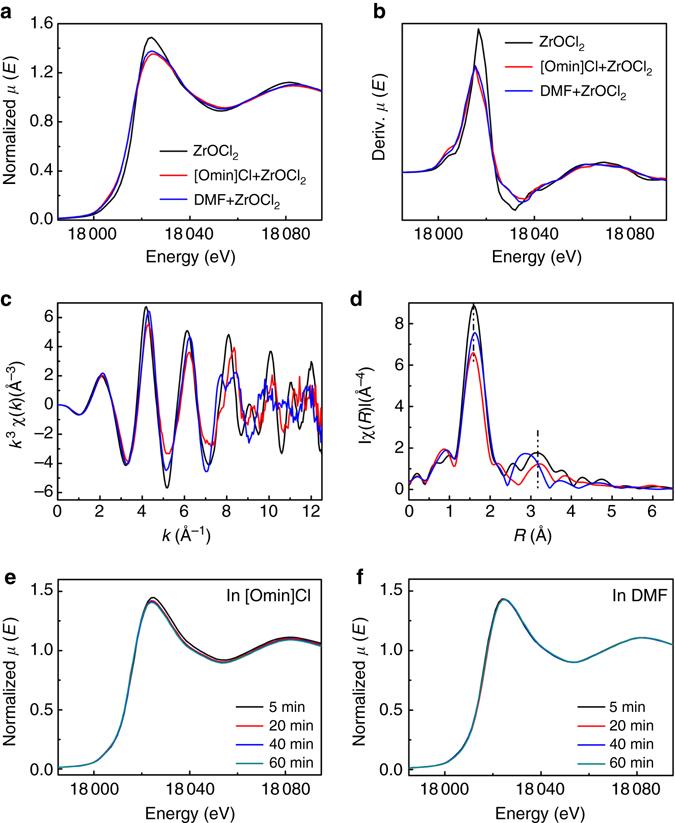
XAFS spectra of ZrOCl_2_ and UiO-66 synthesis mixtures. **a** XANES spectra of solid ZrOCl_2_·8H_2_O and dissolved ZrOCl_2_ in [Omim]Cl and DMF, respectively. **b** Derivated XANES spectra from A. **c** XANES spectra in k-space. **d** XANES spectra in R-space. **e** In situ XANES characterization for the UiO-66 synthesis in [Omim]Cl at 298.2 K with different reaction time. **f** In situ XANES characterization for the UiO-66 synthesis in DMF at 298.2 K with different reaction time

XAFS is of great help in elucidating the local structure of inorganic cornerstones or inorganic backbones for different MOF materials^22, 23, 31^. First of all, the X-ray absorption near edge structures (XANES) of ZrOCl_2_·8H_2_O before and after dissolved in [Omim]Cl and DMF were determined (Fig. 3a). The dissolved ZrOCl_2_ has lower intensity in XANES region (first resonance after the edge) than solid ZrOCl_2_·8H_2_O. Moreover, there is a small increase of the weak pre-edge feature around 18,005 eV for the dissolved ZrOCl_2_, which is further certified by derivated XANES spectra (Fig. 3b). The results manifest that the crystal structure of ZrOCl_2_·8H_2_O is destroyed by solvation. Up to *k* = 7 Å^−1^, the [Omim]Cl and DMF solutions of ZrOCl_2_ perturb the extended XAFS oscillations (Fig. 3c), resulting in a small increase of the oscillation periodicity. It suggests a small shortening of the first shell oxygen ligands. In R-space, the two main peaks corresponding to oxygen and zirconium backscatters at 1.6 and 3.2 Å, respectively, are dramatically reduced and become asymmetric after solvation (Fig. 3d). It is indicative of the decreased coordination number due to the dehydration of zirconyl cation during dissolution. Particularly, the higher R-peak shifts about 0.31 Å for DMF solution, which can be attributed to the dehydration of ZrOCl_2_·8H_2_O crystal and new coordination with DMF.

The X-ray absorption experiments on Zr K-edge were performed in situ for the UiO-66 synthesis in [Omim]Cl and DMF, respectively. As the reaction time is prolonged, there is a decrease for the first resonance after the edge of the reaction system in [Omim]Cl (Fig. 3e). However, no change was observed for this resonance with time of the reaction system in DMF (Fig. 3f). For the UiO-66 synthesis in [Omim]Cl, an evident dehydration of zirconyl cation was observed with reaction time, in other words, the displacement by H_2_BDC linker can easily proceed and the inorganic cornerstones of UiO-66 are gradually formed. However, DMF can form new coordination with ZrOCl_2_ crystal, which would retard the reaction of negatively charged BDC linker and zirconyl cation during UiO-66 synthesis^23^. As a result, a faster reaction takes place in [Omim]Cl than DMF.

### Mechanism

**Fig. 4:**
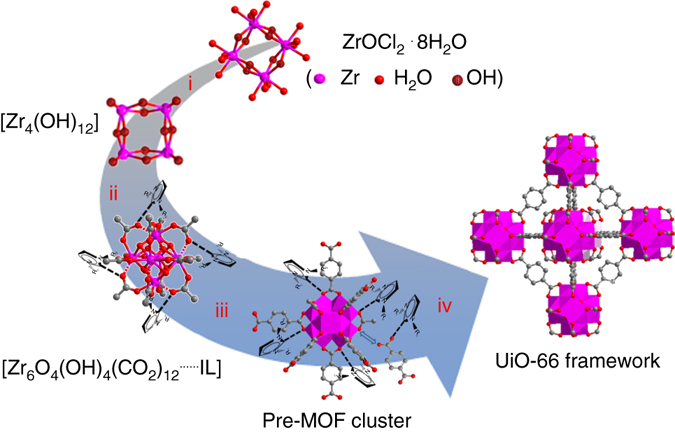
Diagram illustrating the UiO-66 formation in IL. i ZrOCl_2_·8H_2_O hydrolysis to polymeric hydroxide Zr_4_(OH)_12_. ii Complexation of zirconium by addition of HAc. iii IL-assisted linker exchange between HAc and H_2_BDC to produce pre-MOF cluster. iv UiO-66 framework formation

Based on the experimental results, a mechanism for the rapid formation of UiO-66 nanocrystals in IL [C_*n*_mim]X at room temperature is proposed. First, the solvation effect of Zr precursor ZrOCl_2_·8H_2_O causes hydrolysis and dehydration to yield polymeric hydroxide Zr_4_(OH)_12_ (Fig. 4i). The complexation of zirconium by addition of modulator HAc leads to a complex^32^, where the modulator can interact with [C_*n*_mim]X through hydrogen bondings, as evidenced by in-situ ^1^H NMR spectra (Fig. 4ii). Then a linker exchange between the modulators and linkers takes place at the coordination sites of zirconium complex^33–35^. The IL [C_*n*_mim]X plays an important role in this process. Since there are strong interactions (hydrogen bondings) between the modulators and [C_*n*_mim]X, the linker exchange rate is greatly accelerated. In other words, [C_*n*_mim]X promotes the coordination between the linkers and zirconium complex through its strong interactions with the modulator. Consequently, the reaction between the negatively charged BDC linker and zirconyl cation is facilitated and the pre-MOF cluster is produced rapidly in [C_*n*_mim]X at room temperature (Fig. 4iii). Finally, the pre-MOF cluster octahedral edges are bridged by carboxylate groups from the benzenedicarboxylate linker, resulting in the formation of UiO-66 framework (Fig. 4iv).

## Discussion

The present study provides a way for accelerating MOF formation by utilizing IL as solvent. In comparison with the conventional methods for promoting MOF crystallization, e.g., high-energy input or adding additives, the present strategy is rapid, low energy and involves no additional additives. Moreover, the MOF particle size can be easily modulated by changing the anions and cations of ILs. It is expected that this simple route can be applied to the synthesis of different MOFs and MOF composites (e.g., MOF/MOF, metal/MOF, and metal oxide/MOF).

More interestingly, it gives a clue that IL may be used as a cosolvent to the commonly used organic solvent for the rapid MOF synthesis at room temperature. To verify this, the [Omim]Cl/DMF mixtures with different amounts of [Omim]Cl were used as solvents for UiO-66 synthesis at room temperature. The results show that the reaction time can be reduced from 120 h in pure DMF to 48, 32, 10, 4, and 3 h by adding [Omim]Cl with volume fraction of 0.1, 0.2, 0.3, 0.4, and 0.5, respectively (Supplementary Figs. 14–16). The combination of IL and organic solvent will open new perspectives for the rapid and room-temperature synthesis of MOF materials that are largely demanded in chemical industry, particularly from economic and technical points of view.

The room-temperature and rapid synthesis procedure in IL produces MOF nanoparticles with small particle size, missing-linker defects, and large surface area. It is worth noting that the dormant and fully coordinated framework metal ions of MOFs are unavailable for catalysis and many efforts have been devoted to increasing their catalytic activities, such as increasing the number of Lewis and Brönsted acid sites^36^, introducing functional groups at structural defects^37^, etc. Compared with these strategies, the MOF nanocrystals produced in this work involve no second phase or functional groups that usually break or stuck the pore structures of MOF. Therefore, the intrinsic properties of MOF can be well kept to achieve high catalytic activities. The catalytic activities of the as-synthesized UiO-66 for MPV reaction were studied, which is a highly chemoselective reduction method for carbonyl compounds and provides an attractive alternative to H_2_ by using secondary alcohols as hydrogen resources (Supplementary Fig. 17)^38^. The UiO-66 catalysts synthesized in ILs are much more active than that produced by solvothermal method (Supplementary Figs. 18–21). The high catalytic activities of the UiO-66 catalysts synthesized in this work can be ascribed to their unique features. First, in comparison with the MOFs in the form of traditional bulk crystalline, the miniaturization of UiO-66 crystals at nanometer length scale increases the density of catalytic active sites^39, 40^, thus accelerating the reaction. Second, the partially coordinated framework of the as-synthesized UiO-66 is available for catalysis due to the existence of a large number of open metal sites^5, 41^. Third, the large surface area of UiO-66 is favorable for the adsorption of reactants on catalyst, thereby facilitating the catalytic reactivity^5, 42–44^. It is anticipated that the Zr-based MOF nanocrystals synthesized in IL can be utilized as catalysts for other chemical reactions.

## Methods

### MOF synthesis

For the typical synthesis of UiO-66, ZrOCl_2_·8H_2_O (0.4 mmol) and H_2_BDC (0.4 mmol) were added into 10 ml [Omim]Cl, followed by the addition of acetic acid (3 ml). The mixture was stirred at room temperature for a certain time. Methanol was added to wash the as-synthesized crystals twice a day for 2 days to remove [Omim]Cl and the unreacted precursors. The precipitate was collected by centrifugation and dried under vacuum at 60 °C for 24 h. The yield for UiO-66 preparation in [Omim]Cl was 82%. The IL after use can be renewed by rotary evaporation due to its low vapour pressure. For the synthesis of other MOFs, the organic ligand was changed and the other experimental conditions were the same with those above. For comparison, UiO-66 was synthesized in DMF at room temperature by the procedure similar to that in IL.

### MOF characterization

XRD was performed on X-ray diffractometer (Model D/MAX2500, Rigaka) with Cu-Kα radiation and the scan speed 5 min^−1^. The morphologies were characterized by TEM JEOL-1011. FT-IR spectra were determined by Bruker Tensor 27 spectrometer and the sample was prepared by KBr pellet method. TGA measurement was carried out with a heating rate of 10 °C min^−1^ using PerkinElmer Pyris 1 under air flow of 20 ml min^−1^. X-ray photoelectron spectroscopy was determined by ESCALab220i-XL electron spectrometer from VG Scientific using 300 W AlKα radiation. The base pressure was 3 × 10^−9^ mbar. The binding energies were referenced to C1s line at 284.8 eV from adventitious carbon. The porosity properties were gained from N_2_ adsorption-desorption isotherms at 77 K using a Quadrasorb SI-MP system. Prior to analysis, the sample was activated at 150 °C for 12 h on a Micrometrics Smart VacPrep System. The elemental contents of C, H, and N in UiO-66 were determined using Flash EA1112 from Thermo. The Zr content in UiO-66 was determined by inductively coupled plasma-atomic emission spectrometry method (VISTA-MPX).

### SAXS, XAFS, and ^1^H NMR on MOF formation

The SAXS experiment was carried out at Beamline 1W2A at Beijing Synchrotron Radiation Facility (BSRF). The wavelength was 1.38 Å and the distance of sample to detector was 2.10 m. The XAFS experiment was carried out at Beamline 1W1B at BSRF. Data of XAFS were processed using the Athena and Artemis programs of thee IFEFFIT package based on FEFF 6. Prior to merging, the spectra were aligned to the first and largest peak in the smoothed first derivative of the absorption spectrum, the background was removed, and the spectra were processed to obtain a normalized unit edge step. Data were processed with k^3^-weighting and an Rbkg value of 1.0. Merged data sets were aligned to the largest peak in the first derivative of the adsorption spectrum. Normalized XANES data were obtained directly from the Athena program of the IFEFFIT package. Moreover, the reaction process was monitored by ^1^H NMR (Bruker Avance III 600 HD spectrometer).

### Data availability

All data is available from the authors upon reasonable request.

## Electronic supplementary material

Supplementary Information
