# Supplementary material for: Ionic liquid accelerates the crystallization of Zr-based metal–organic frameworks
Source: Nat Commun. 2017 Aug 2;8:175. doi: 10.1038/s41467-017-00226-y (PMC5539316; doi:10.1038/s41467-017-00226-y)
Supplement: Supplementary file 1 — Supplementary Information [file 41467_2017_226_MOESM1_ESM.pdf]

Supplementary Methods

Materials. 1-hexyl-3-methylimidazolium chloride ([Hmim]Cl), 1-octyl-3-methylimidazolium chloride ([Omim]Cl), 1-decyl-3-methylimidazolium chloride ([Dmim]Cl), 1-octyl-3-methylimidazolium bromide ([Omim]Br) and 1-octyl-3-methylimidazolium iodide ([Omim]I) were provided by Lanzhou Greenchem ILS, LICP, CAS (>98% purity). Zirconium oxychloride octahydrate ($ZrOCl_2\cdot 8H_2O$), N,N-dimethylformamide (DMF), methanol, acetic acid, terephthalic acid (H_2BDC), 2-hydroxyterephthalic acid (H_2BDC-OH), 2-aminoterephthalic acid (H_2BDC-NH_2), 2-nitroterephthalic acid (H_2BDC-NO_2), biphenyl-4,4′-dicarboxylate (H_2BPDC) and 2,2′-bipyridine-5,5′-dicarboxylic acid (H_2BPYDC) were all purchased from Sigma-Aldrich.

Data analysis on N_2 adsorption-desorption experiment. The data were analyzed using the Quantachrome ASIQwin v2.0 software (Quantachrome Instruments). Brunauer-Emmet-Teller (BET) surface areas were calculated according to the consistency criteria outlined as previously reported[1]. The BET equation is as follows:

$$\frac{P/P_0}{n(1 - P/P_0)} = \frac{1}{n_m C} + \frac{C-1}{n_m C}(P/P_0) \quad (1)$$

P/P_0 is the relative pressure, n is the amount of gas adsorbed, n_m is the amount of gas necessary to form a monolayer on the adsorbent and C is the BET constant.

Catalytic test. Catalytic reactions were carried out in 20 mL stainless steel batch reactor with a magnetic stirring bar. For Meerwein-Ponndorf-Verley (MPV) reductions, 4-tert-butylcyclohexanone (0.6 mmol; 99%, Aldrich) and isopropanol (2 mL; 99.5 %, Aldrich) were loaded in the reactor. N-decane was added as an internal standard. For each catalyst, 25 mg MOF was used. The vessel was placed in an aluminium heating block at 130 °C and stirred for a desired time. After reaction for a certain time, the catalyst was separated and the product was analyzed by gas chromatograph (Agilent 6820).

Supplementary Figures

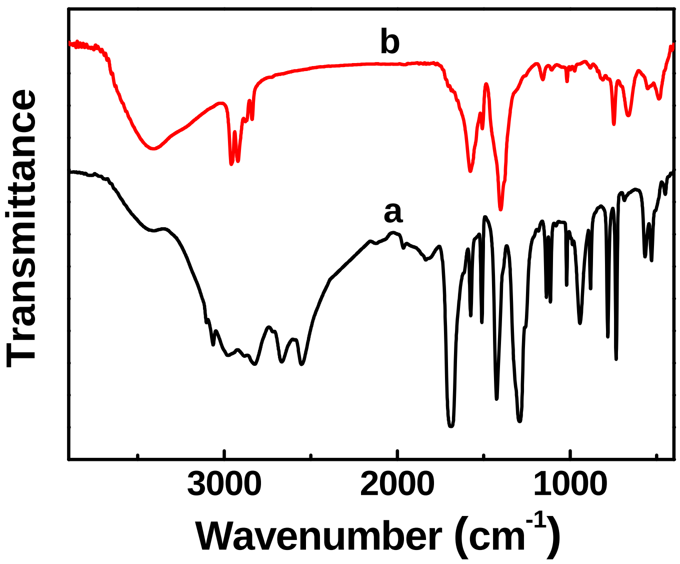

Supplementary Figure 1. FT-IR spectra of H_2BDC (a) and UiO-66 (b). Compared with the FT-IR spectrum of H_2BDC, the wavenumber difference between asymmetric and symmetric vibration of carboxylate anions is narrowed, indicating that the carboxylate groups of H_2BDC are coordinated to Zr (IV) ions.

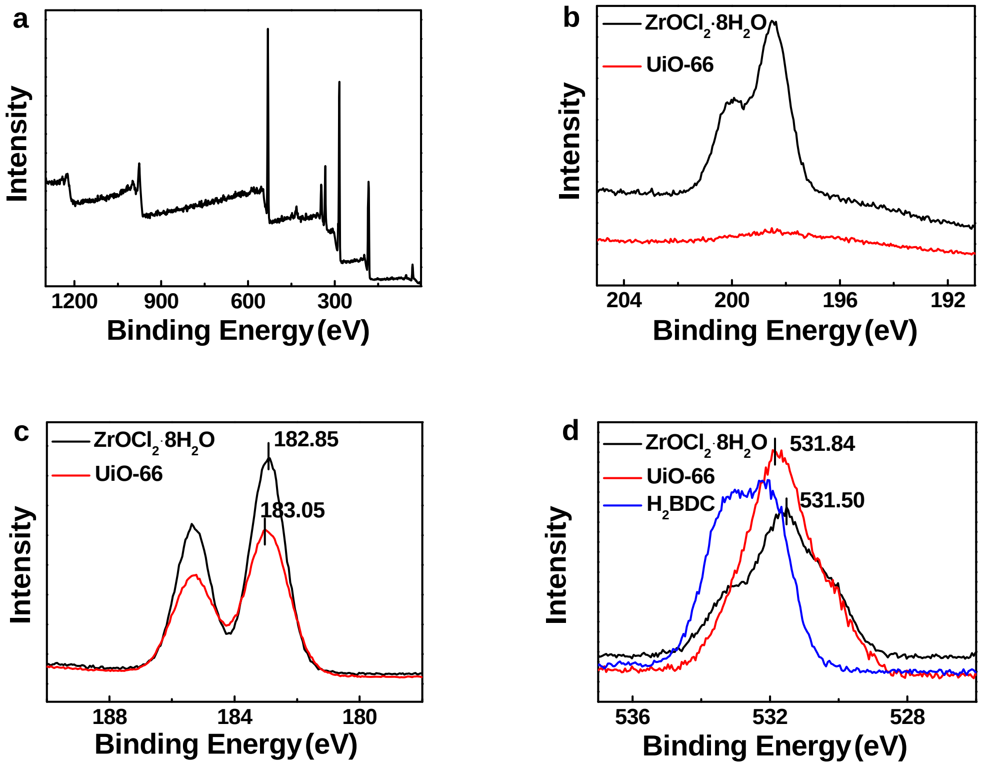

Supplementary Figure 2. XPS spectra of the UiO-66 synthesized in [Omim]Cl. (a) A wide-range XPS spectrum; (b) Cl2p spectra; (d) Zr3d spectra; (d) O1s spectra. The absence of N1s and Cl2p peaks proves that there is no IL left in the UiO-66 nanocrystals. The binding energy of Zr in UiO-66 (183.05 eV) is higher than that of $ZrOCl_2$ (182.85 eV), while the binding energy of O1s in UiO-66 is between those of H_2BDC and $ZrOCl_2$. The result indicates the formation of framework.

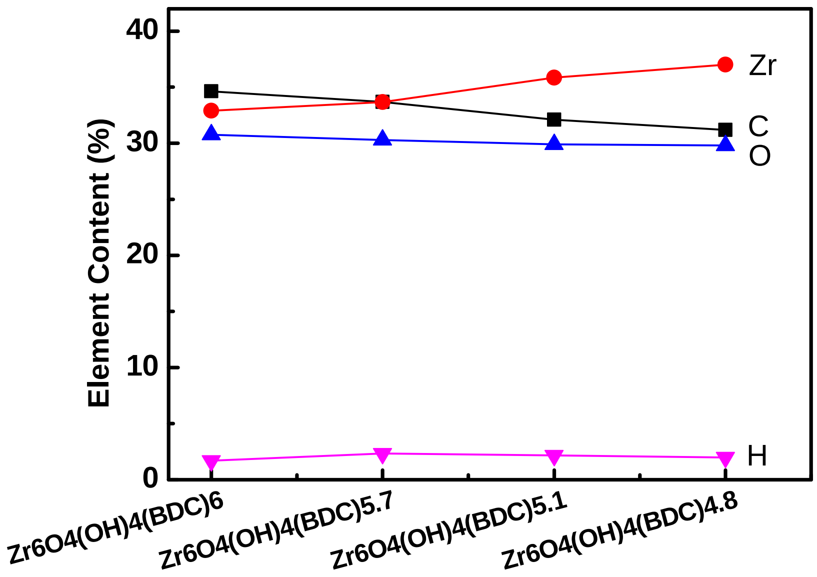

Supplementary Figure 3. Element contents of Zr (red), C (black), O (blue) and H (magenta) in the standard UiO-66 ($Zr_6O_4(OH)_4(BDC)_6$) and the $Zr_6O_4(OH)_4(BDC)_{5.7}$, $Zr_6O_4(OH)_4(BDC)_{5.1}$, $Zr_6O_4(OH)_4(BDC)_{4.8}$ synthesized in [Hmim]Cl, [Omim]Cl and [Dmim]Cl, respectively. There is more zirconium and less carbon content in the UiO-66 synthesized in ILs than the standard UiO-66 ($Zr_6O_4(OH)_4(BDC)_6$), which is consistent with a decrease in the amount of linkers. The Zr/C content ratio for the three UiO-66 increases in order of [Hmim]Cl<[Omim]Cl<[Dmim]Cl, indicating the presence of more missing-linker defects of the UiO-66 synthesized in IL with longer alkyl chain. The element N was not detected, proving the absence of IL in the product.

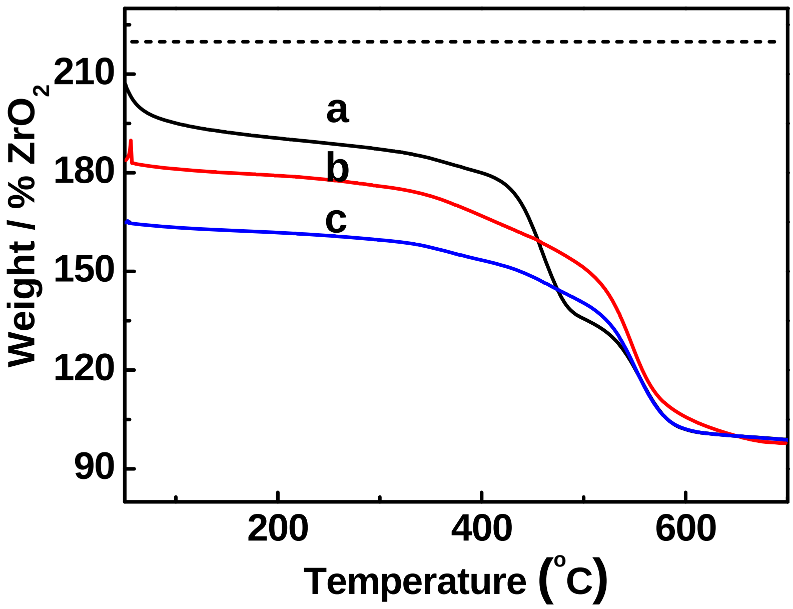

Supplementary Figure 4. TG curves of the UiO-66 synthesized in [Hmim]Cl (a), [Omim]Cl (b) and [Dmim]Cl (c). The molecular weight of $Zr_6O_6(BDC)_6$ is a factor of 2.2 higher than $6ZrO_2$, the only solid product. Thus, if the end weight of the TG run is normalized to 100%, the plateau (representing the empty, solvent free material) should ideally reach 220%. However, it typically falls, meaning that some of the linkers are missed from the framework[2].

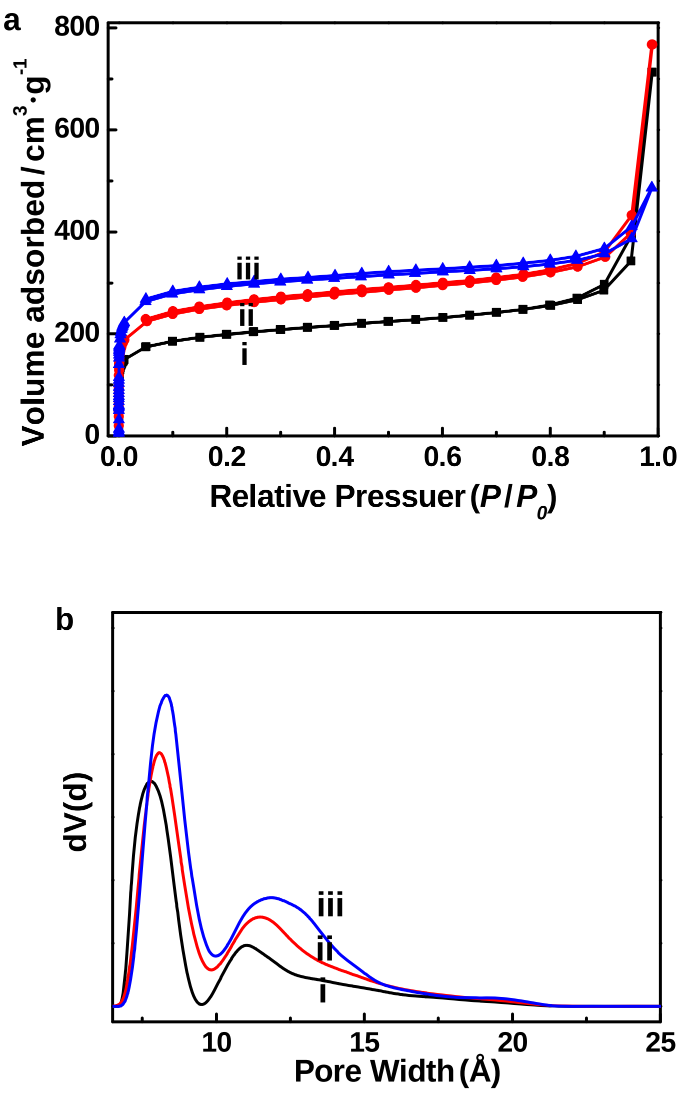

Supplementary Figure 5. (a) N_2 adsorption-desorption isotherms and (b) pore-size distributions of the UiO-66 synthesized in [Hmim]Cl (i), [Omim]Cl (ii) and [Dmim]Cl (iii). The surface area of UiO-66 synthesized by solvothermal process is 1187 $m^2 \cdot g^{-1}$ with a pore volume 0.520 $cm^3 \cdot g^{-1}$ in DMF[3], while the surface areas are 1309, 1519 and 1603 $m^2 \cdot g^{-1}$ for the UiO-66 synthesized in [Hmim]Cl, [Omim]Cl and [Dmim]Cl, with the pore volume 0.589, 0.647 and 0.763 $cm^3 \cdot g^{-1}$, respectively. The pore-size distribution curves show that the micropore size is enlarged as the ILs alkyl chain length increases. It has been reported that a direct consequence of an increased number of defects is the formation of larger micropores[4], resulting in larger pore volume and more accessible space.

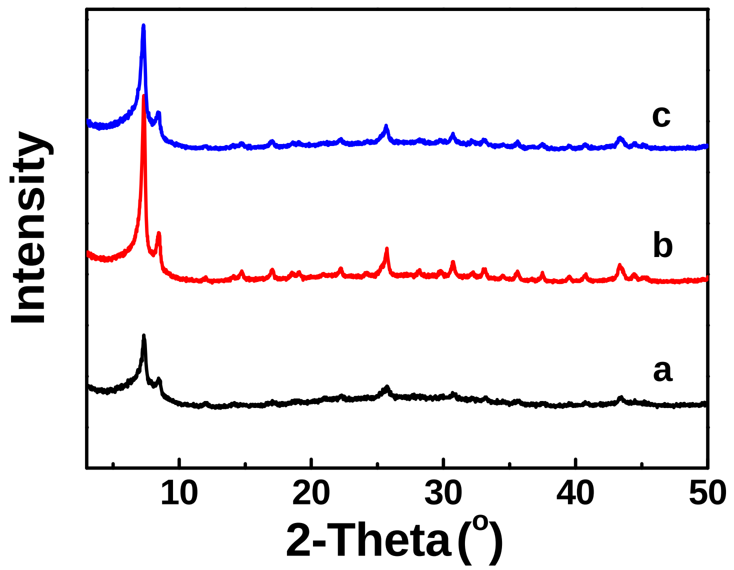

Supplementary Figure 6. XRD patterns of the samples synthesized in [Hmim]Cl with different reaction time: (a) 0.5 h, (b) 1 h, (c) 6 h.

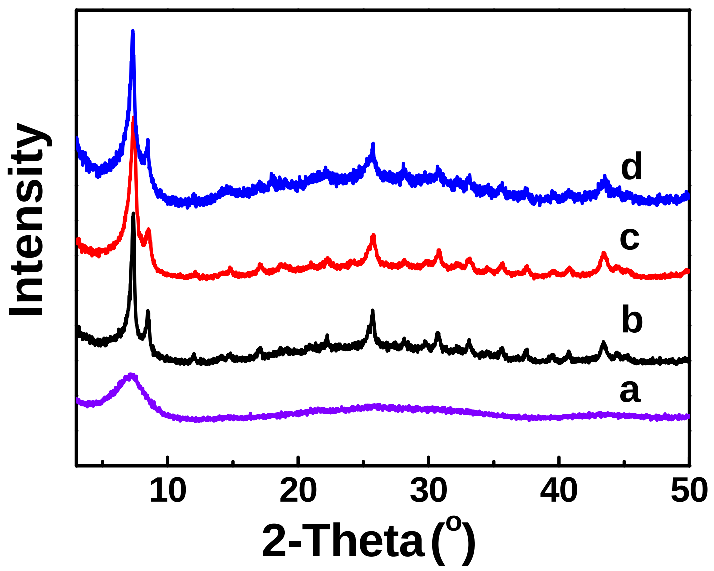

Supplementary Figure 7. XRD patterns of the samples synthesized in [Dmim]Cl with different reaction time: (a) 1 h, (b) 2 h, (c) 6 h, (d) 12 h.

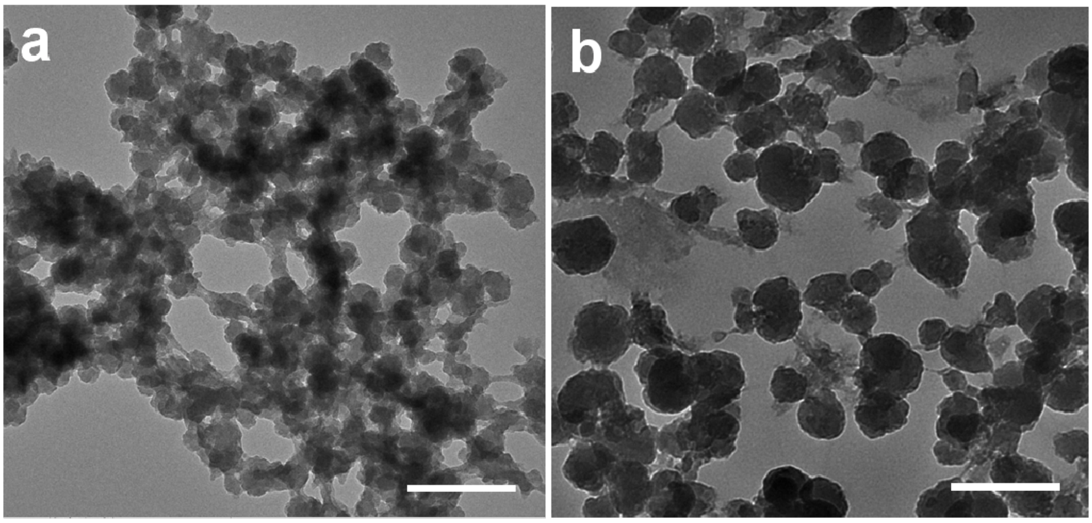

Supplementary Figure 8. TEM images of the UiO-66 synthesized in [Hmim]Cl (a) and [Dmim]Cl (b). Scale bars, 200 nm.

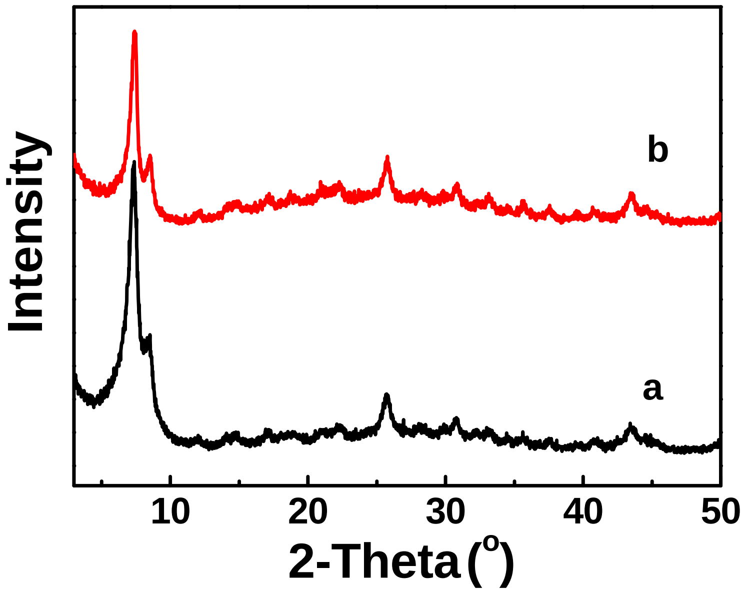

Supplementary Figure 9. XRD patterns of the samples synthesized in [Omim]Br with a reaction time of 6 h (a) and in [Omim]I of 18 h (b).

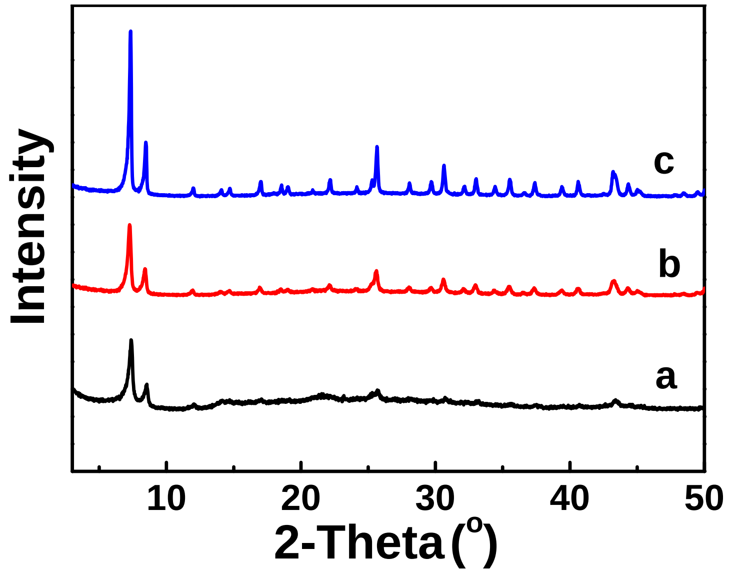

Supplementary Figure 10. XRD patterns of the UiO-66-X synthesized in [Omim]Cl: (a) UiO-66-NH_2, (b) UiO-66-NO_2, (c) UiO-66-OH.

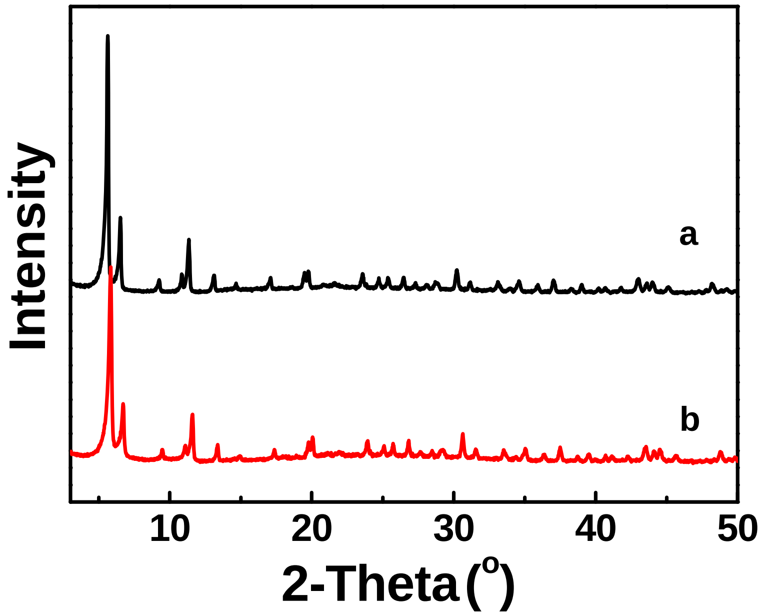

Supplementary Figure 11. XRD patterns of the Zr-MOFs synthesized in [Omim]Cl: (a) UiO-67, (b) Zr-MOF-bpydc.

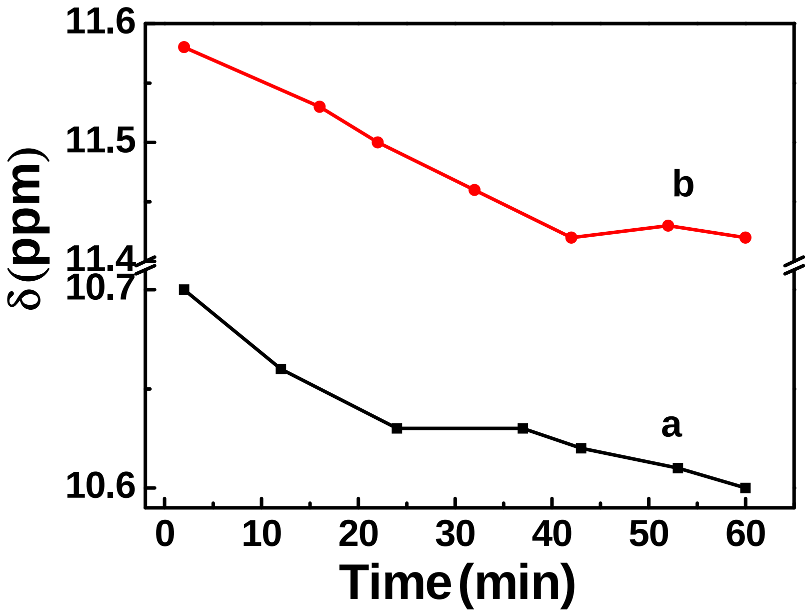

Supplementary Figure 12. Chemical shift (δ) of 2#H with different reaction time in [Hmim]Cl (a) and [Dmim]Cl (b).

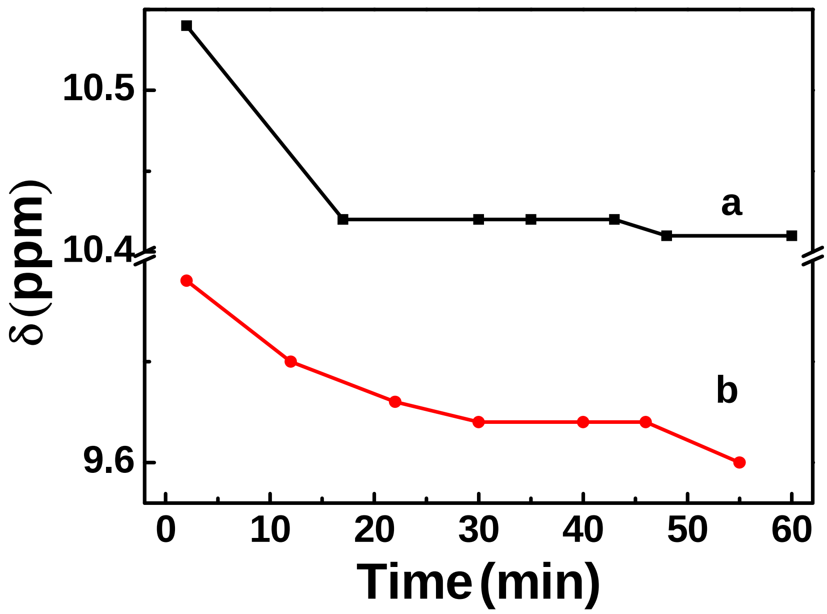

Supplementary Figure 13. Chemical shift (δ) of 2#H with different reaction time in [Omim]Br (a) and [Omim]I (b).

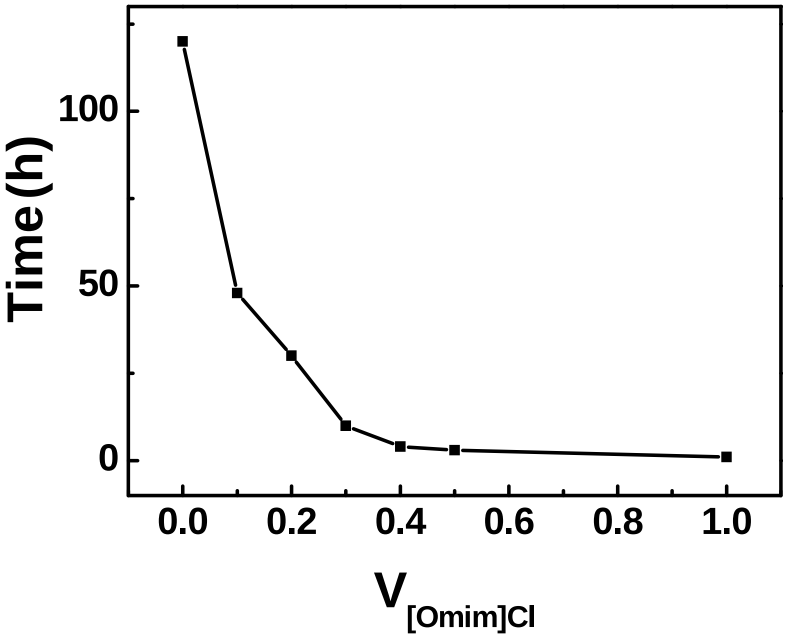

Supplementary Figure 14. Time used for UiO-66 formation in [Omim]Cl/DMF mixtures with different volume fraction of [Omim]Cl ($V_{[Omim]Cl}$).

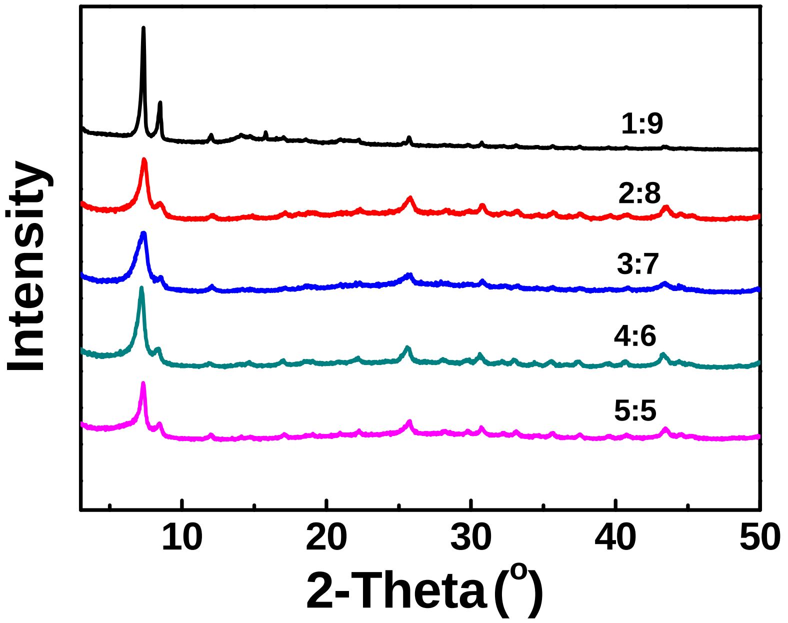

Supplementary Figure 15. XRD patterns of the UiO-66 synthesized in [Omim]Cl/DMF mixtures with different volume ratio of [Omim]Cl to DMF.

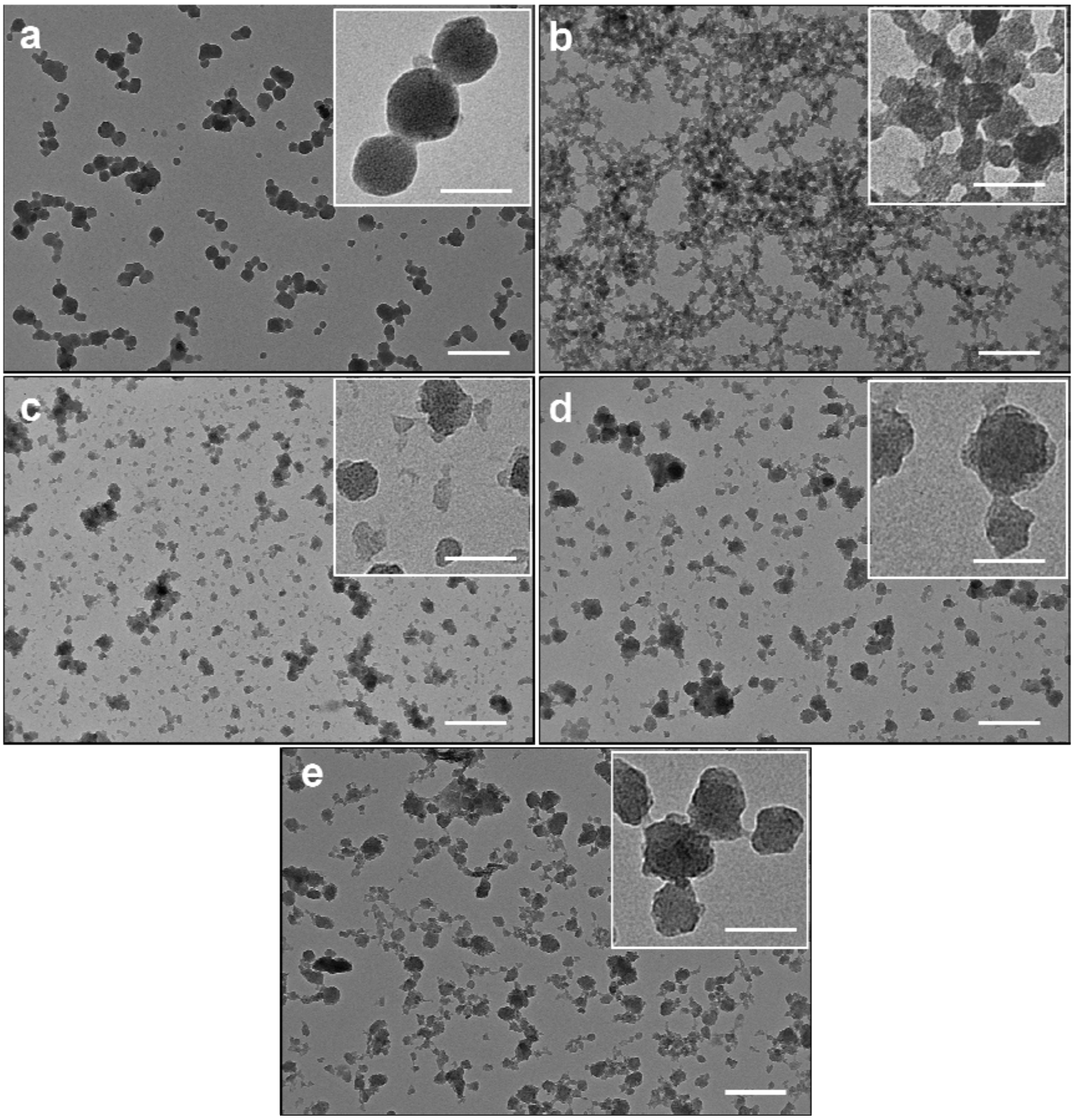

Supplementary Figure 16. TEM images of the UiO-66 synthesized in [Omim]Cl/DMF mixtures with [Omim]Cl volume fraction of 0.1 (a), 0.2 (b), 0.3 (c), 0.4 (d) and 0.5 (e). Scale bars, 200 nm. The insets show the magnified TEM images, scale bars, 50 nm.

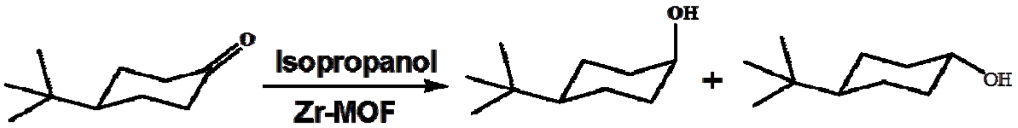

Supplementary Figure 17. MPV reaction of TCH with isopropanol. A solvent-free procedure was applied for the MPV reaction of 4-tert-butylcyclohexanone (TCH) by using isopropanol (IPA) as both hydrogen donor and solvent.

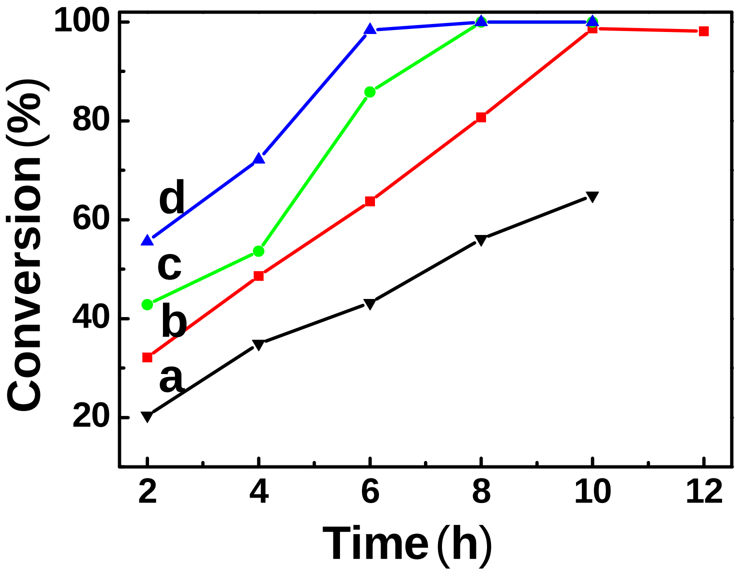

Supplementary Figure 18. Conversion of TCH over UiO-66 synthesized through a standard solvothermal process (a) and synthesized in [Hmim]Cl (b), [Omim]Cl (c) and [Dmim]Cl (d) (solvent-free, 130 °C, TCH:IPA:Zr^{4+} = 10:50:1). Clearly, the catalytic activities of the UiO-66 catalysts synthesized in ILs are higher than that produced by solvothermal method. For example, as catalyzed by the UiO-66 synthesized in [Dmim]Cl, TCH can be completely converted at 6 h (curve d), while the conversion of TCH over the UiO-66 synthesized by solvothermal route is only 43.2% at the same reaction time (curve a).

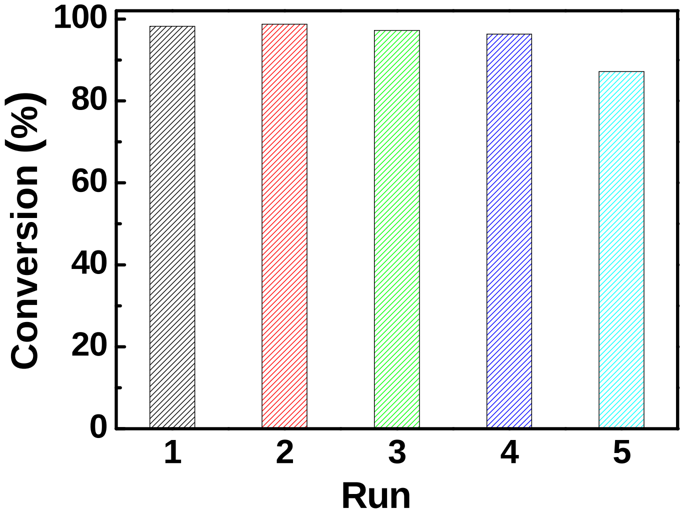

Supplementary Figure 19. Reusability of the UiO-66 synthesized in [Dmim]Cl. The reaction time for each run is 6 h.The catalyst shows no evident drop of catalytic activity after four runs and the conversion decreases to 87% in the fifth run. The declined catalytic activity of UiO-66 after used for five runs can be attributed to the partially blocked micropores, as revealed by the N_2 adsorption-desorption isotherms shown in Supplementary Figure 20.

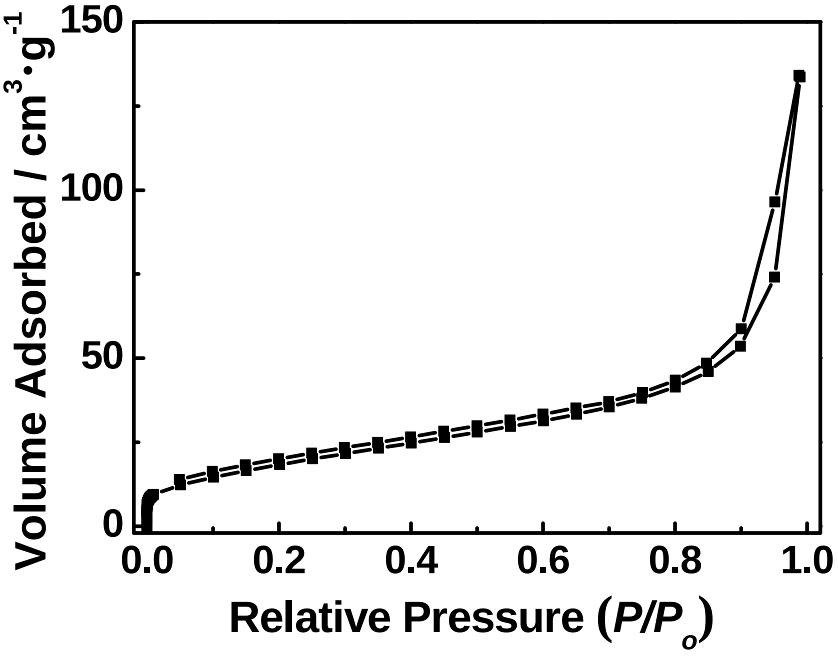

Supplementary Figure 20. N_2 adsorption-desorption isotherms of the UiO-66 after used for five runs.

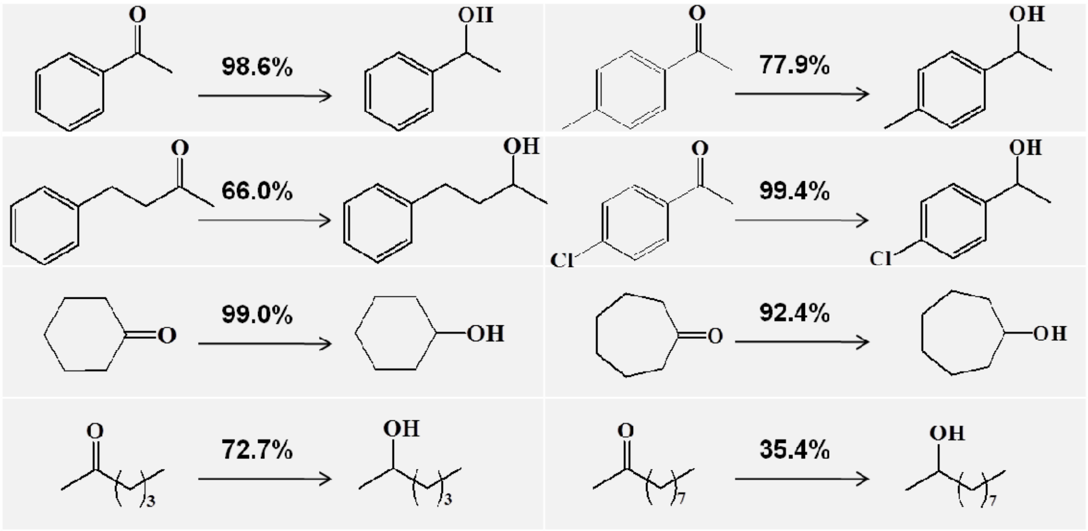

Supplementary Figure 21. Conversions of a series of MPV reactions catalyzed by the UiO-66 synthesized in [Omim]Cl. The reaction time was 10 h and the other experimental conditions were the same as above. The UiO-66 shows excellent catalytic activity for the conversion of the examined compounds. For the reduction of acetophenone, the conversion to 1-phenethyl alcohol can reach 98.6%. The conversion for the carbonyl compound with a benzene group decreases with the substitution of $-CH_3$ in para-position (77.9%) or the increasing alkyl chain length (66.0%), while increases with the replacement of -H by -Cl in para-position (99.4%). It can be ascribed to the electron effect that the substituent group able to reduce the electron density of reactant may be in favour of reduction. Therefore, the electron-withdrawing group (-Cl) is favorable for the reaction[5]. Further, UiO-66 can catalyze the reduction of cyclohexanone and cycloheptanone with high conversions of 99.0% and 92.4%, respectively. The linear ketones of 2-hexanone (conversion 72.7%) and 2-heptanone (conversion 35.4%) exhibit lower reactivity as compared with those of cyclic ketones. It suggests that the steric hindrance of reactant is disadvantageous for the transformation to corresponding alcohols[6].

Supplementary References

1. DeStefano, M. R. *et al.* Room temperature synthesis of UiO-66 and thermal modulation of densities of defect sites. *Chem. Mater.* **29,** 1357-1361 (2017).
2. Shearer, G. C. *et al*. Tuned to perfection: Ironing out the defects in metal-organic framework UiO-66. *Chem. Mater.* **26,** 4068-4071 (2014).
3. Cavka, J. H. *et al*. A new zirconium inorganic building brick forming metal organic frameworks with exceptional stability. *J. Am. Chem. Soc*. **130,** 13850-13851 (2008).
4. Katz, M. J. *et al*. A facile synthesis of UiO-66, UiO-67 and their derivatives. *Chem. Commun*. **49,** 9449-9451 (2013).
5. Wang, D. *et al*. Sodium hydroxide-catalyzed transfer hydrogenation of carbonyl compounds and nitroarenes using ethanol or isopropanol as both solvent and hydrogen donor. *J. Mol. Catal. A: Chem*. **400,** 14-21 (2015).
6. Song, J. *et al*. Porous zirconium-phytic acid hybrid: A highly efficient catalyst for Meerwein-Ponndorf-Verley reductions. *Angew. Chem., Int. Ed*. **127,** 9531-9535 (2015).
